# Autothermal Thermophilic Aerobic Digestion (ATAD) for Heat, Gas, and Production of a Class A Biosolids with Fertilizer Potential

**DOI:** 10.3390/microorganisms7080215

**Published:** 2019-07-25

**Authors:** J. Tony Pembroke, Michael P. Ryan

**Affiliations:** Department of Chemical Sciences, School of Natural Sciences and Bernal Institute, University of Limerick, V94 T9PX Limerick, Ireland

**Keywords:** ATAD, Class A biosolids, digestion

## Abstract

Autothermal thermophilic aerobic digestion (ATAD) is a microbial fermentation process characterized as a tertiary treatment of waste material carried out in jacketed reactors. The process can be carried out on a variety of waste sludge ranging from human, animal, food, or pharmaceutical waste where the addition of air initiates aerobic digestion of the secondary treated sludge material. Digestion of the sludge substrates generates heat, which is retained within the reactor resulting in elevation of the reactor temperature to 70–75 °C. During the process, deamination of proteinaceous materials also occurs resulting in liberation of ammonia and elevation of pH to typically pH 8.4. These conditions result in a unique microbial consortium, which undergoes considerable dynamic change during the heat-up and holding phases. The change in pH and substrate as digestion occurs also contributes to this dynamic change. Because the large reactors are not optimized for aeration, and because low oxygen solubility at elevated temperatures occurs, there are considerable numbers of anaerobes recovered which also contributes to the overall digestion. As the reactors are operated in a semi-continuous mode, the reactors are rarely washed, resulting in considerable biofilm formation. Equally, because of the fibrous nature of the sludge, fiber adhering organisms are frequently found which play a major role in the overall digestion process. Here, we review molecular tools needed to examine the ATAD sludge consortia, what has been determined through phylogenetic analysis of the consortia and the nature of the dynamics occurring within this unique fermentation environment.

## 1. Introduction

ATAD (autothermal aerobic thermophilic digestion) is a liquid composting, tertiary sludge treatment process which utilizes the metabolic heat generated by way of microbial digestion to stabilize the treated sludge and effectively pasteurize the digestate [1,2,3]. This produces class A biosolids suitable for land spread. The ATAD process has been utilized to treat a variety of waste streams (Table 1) including animal waste, food, brewery slaughterhouse wastes and domestic wastes. It has been demonstrated that ATAD can be used to process a variety of high strength waste streams at elevated temperatures with biological oxygen demand (BOD) reduction of 95% and chemical oxygen demand (COD) reductions up to 99% [4,5]. The process can operate as a one-stage system [6] or as a two-stage system [7] that incorporates a mesophilic stage and then follow on thermophilic stage. Generally, the sludge itself is thickened via polymer addition to 7–8% total solids followed by aeration in jacketed bioreactors, where the addition of air and mixing causes biodegradation to proceed with the energy converted to biomass and heat. Because of the jacketed nature of the bioreactors, the trapped heat results in a rise in temperature to above 60 °C, often reaching up to 75 °C [8]. The thermal processing results in a sanitized digestate [9] where pathogens, carried over from the initial secondary sludge, are inactivated. This results in a Class A Biosolids, which has the potential for land spread as a liquid fertilizer. During the process, stages deamination of proteinaceous material results in ammonia release [7]. This results in the case of domestic wastes, in a rise in pH to above 8. This increased pH has been proposed to allow better reactor performance, higher enzyme activity, facilitates oxidation, accelerates humification but limits microbial diversity by the unique combination of pH and temperature [10]. These conditions provide a unique environmental niche with a distinctive nutrient profile of often somewhat recalcitrant biological material that results from primary and secondary treatment waste processes the nature of which varies according to waste type (Table 1).

In addition, the elevated slightly alkaline pH coupled to the elevated temperature also selects a unique microbial population that will be discussed below. Many of the large-scale reactors and their operating parameters (mixing, air addition, sludge additions and removal in semi continuous mode) are imperfect in terms of providing optimal aerobic conditions. This is due to low oxygen solubility at elevated temperatures, low dissolved oxygen concentration because of the high level of microbial degradation at the thermophilic stage, imperfect mixing and aeration to account for these changes, biofilm formation on the reactor surfaces, and the sludge viscosity [4,7]. Due to a combination of these conditions, although termed aerobic digestion, there are many reports of the recovery and activity of anaerobes, which undoubtedly play a major part in the ATAD process [2,3,14,19]. The process is also dynamic from a microbial perspective with various transitions occurring at various stages during the digestion. Initially, mesophiles dominate and degrade the more readily digestible substrates. Then, as the pH and temperature rises, the mesophilic population is replaced by a more thermoduric and thermophilic population adapted for growth at slightly alkaline thermophilic temperatures [20]. The microbial diversity operating within ATAD reactors is key to the degradation and stabilization of the sludge substrate and the maintenance of thermophilic conditions [2,20]. This diversity has been examined for several different ATAD processes such as swine waste [21], pharmaceutical waste [17], and domestic sludge [2,3,19,22,23]. Previous studies attempted to utilize culture-based methods [24] to identify the nature of the microbial diversity, but these proved to be limiting [25], thereby necessitating the use of molecular tools to definitively address the diversity present in ATAD reactors.

## 2. Limitations of Culture-Based Techniques in Determining ATAD Microbial Diversity

In attempts to optimize and understand the ATAD process, it is important to know the nature of the microbial diversity present and the metabolic activities this community undertakes. Initial studies focusing on ATAD communities applied culture-based techniques [11,24,26,27,28] and revealed the prevalence of thermophilic Bacillus species in the main. However, microscopic analysis revealed a much greater diversity and established that culture-based techniques applied to ATAD did not reveal the diversity present [29]. Indeed, it was established that culture-based techniques select for rapidly growing aerobic heterotrophs able to adapt to the culturing environment and clearly do not reflect the diversity present [29,30]. Many of these early studies identified problems and issues with culture-based methodology relating to ATAD including sludge sampling and inocula preparation, adhesion of microbes to fibers and particle surfaces, microorganism extracellular polysaccharide associations, microcolony formation, biofilms on reactor surfaces and viable non-culturable organisms [31,32,33]. Various optimization techniques were attempted including attempts to disperse microorganisms prior to culture via sonication, combinations of chemical treatments however, these disaggregation techniques are sludge dependent and proved of limited use [34]. Indeed, various attempts to vary culture pH, oxygen potential, enumeration media, temperature, and time of incubation have also been attempted [35], but major limitations were discovered. In the case of thermophilic communities, the need for complex nutrients and trace elements [36] complicates the culture process even more while issues such as microbial synergism and symbiosis, which are known to be a feature of ATAD [2], add further layers of complexity. Culture-based techniques were utilized to recover viable organisms from an ATAD treating a mixed domestic and meat waste ATAD [19]. From pure culture, 16 of 17 strains were identified as *Geobacillus* most closely related to *Geobacillus thermodenitrificans* or related to *Geobacillus* strains isolated previously from compost. In addition, strains related to Thermus were also isolated, the first time this organism was reported [19]. Thus, the inability to design culture regimes that take all potential variables into account make recovery of all microorganisms present in a complex ATAD community impossible [37] and hence the reliance on molecular based technology.

## 3. Sampling of ATAD Diversity

The optimal determination of molecular diversity in an ATAD process, or indeed in any niche, relies heavily on the proper preparation and processing of microbial DNA from the sampling site. This target DNA is the key element, which is followed by subsequent analysis of the DNA by techniques such as 16S rDNA sequencing, denaturing gradient gel electrophoresis (DGGE) or sequencing for metagenome analysis. The validity of the subsequent phylogenies or dynamics of the populations recovered is highly dependent on the optimum recovery of the DNA. In attempting to carry out an analysis of the microbial diversity of a large scale ATAD reactor (110 m^3^) treating mixed domestic waste, a number of key limitations in the sampling and processing were identified [9]. These were not apparent in the analysis of more traditional sampling sites but were key to the proper analysis of the ATAD niche. Initial attempts to recovery the diversity of the microbial population indicated there were a number of factors that limited recovery. An extensive analysis of the conditions that limited diversity recovery was carried out in an attempt to optimize the identification of the key microbial players that participated in the ATAD process, particularly at the elevated temperatures [9,20,38]. During the various transition phases from mesophilic to thermophilic conditions, and also during the thermophilic stages, significant amounts of lysis were observed as the population transitioned to the increased temperatures or more alkaline conditions [33]. This resulted in the release of large amounts of nucleases with varied substrate specificities, many of which were thermostable and capable of interfering with the recovery of template DNA for subsequent phylogenetic analysis [20,38]. Additionally, this lead to the liberation of a diverse range of proteases, many of which were also thermostable were found to dramatically effect subsequent DNA amplification if not inhibited or removed during the initial DNA extraction protocols. Inhibition or removal of these activities was shown to be a key element of diversity recovery [20]. The presence of humic substances in the sludge was also shown to be inhibitory to a number of polymerases used to amplify templates for 16S rDNA analysis as were the choice of 16S primers, with variation shown in their ability to amplify the 16S rDNA target [38]. Analysis also determined that extraction methodology, choice of polymerase, and the use of additives during processing of DNA samples were all key to the optimal and reproducible recovery of ATAD microbial diversity [20,38]. Other strategies for optimizing recovery of environmental DNA were also examined, with many appearing in the literature these include serial dilution of the template [39], however this resulted in dilution also of rare templates which effects diversity recovery. Addition of a range of chemical additives [40] has also been proposed and trialed on ATAD DNA recovery [38], but these also depend on the nature of the inhibitory substances that might be present in ATAD samples. To verify the validity of ATAD DNA extracts to optimize diversity recovery three criteria used [20], the recovery of high molecular weight DNA, the absence of PCR inhibitors, and extracts that recover all possible genomes. A number of DNA extraction protocols were initially compared, spike and recovery of known templates was carried out, the quality of the DNA analyzed and the reactivity of the extracted template DNA to a variety of DNA polymerases and 16S rDNA primers analyzed to optimize the recovery and subsequent molecular analysis of ATAD DNA [18]. Examination indicated that a number of factors associated with ATAD sludge caused interference with optimal recovery of DNA. These included the presence of humic substances in ATAD sludge, where even small quantities have been shown to inhibit DNA polymerase activities [41]. The presence of nucleases, which are liberated during lysis as the temperatures increase, needed addition of EDTA at 5 mM (some five times recommended in buffers) and formamide, and heat treatment of the extracted DNA was necessary to limit template and even primer degradation [14,20,38]. Dilution of template DNA was found to relieve PCR inhibition but this had a major effect on diversity recovery. Neither did the efficiency of PCR recovery correlate with the length of the PCR template. A number of 16S rDNA primers were trialed, but no correlation was detected with recovery and amplicon length. It was observed that use of V3-V3 and V1-V19 16S rDNA primer sets were more resistant to inhibition with ATAD extracts, and these were routinely utilized [20]. Given that many other ATAD substances, in addition to humic substances, present in ATAD sludge such as carbohydrates and fiber could affect amplification a number of adjuvants were also utilized in attempts to optimize amplification. It was observed that the addition of bovine serum albumen (BSA), (which may bind polyphenols and humic substances and act as alternative substrates for residual extracted ATAD proteases) and formamide (weakens hydrogen bonds between nucleotides reducing complex formation and enhances specificity, possesses some DNase inhibitory activity) resulted in better DNA recovery for further analysis. Spiked additions of diluted, known templates were added periodically as a control to the ATAD extracts to determine the ability of the optimized techniques to recovery ‘rare’ (diluted) spiked DNA [20]. This aided in the validation of the various adjuvant additions for optimizing the template purification strategy. In summary, key elements identified [20,38] were that specific modifications to extraction and amplification reactions were necessary to optimize recovery of templates for molecular analysis of ATAD sludge microbial communities. The Power Soil DNA isolation kit (Qiagen, Manchester, UK) was found to give the best extraction coupled to the modifications specific to ATAD as outlined above [20]. A number of other key elements were also identified, such as processing the samples immediately following sampling as storage or transport was found to limit template DNA recovery, while maintaining DNA in 1% formamide was essential for longer-term storage. Thus, when attempting to determine the true community diversity of an unusual niche, such as ATAD at elevated temperatures, optimizing the preparation protocols is an essential element in recovery of this diversity.

## 4. PCR-DGGE Based Molecular Methodology to Evaluate ATAD Diversity

In examining the operation and stability of an ATAD process, it is important to examine the community structure and diversity of species present as a function of the process stage. As reaching autothermal conditions is a key element of the process to pasteurize sludge from a health and safety perspective. The thermophilic communities that drive the process to reach these temperatures are not only essential but also their stability for long-term operation of the process is a key element of ATAD. As the ATAD process proceeds, there are changes in oxygen availability (as temperature rises, solubility decreases and there is more biomass utilization), nutrients (as biodegradation proceeds towards more recalcitrant substrates during the process), pH (as ammonia is released during deamination), and most especially in temperature (as the microbial degradation leads to heat evolution which is essentially trapped via the reactor design). The various community transitions and microbial population changes in response to these process variations are potentially key elements to the stability and functioning of the entire ATAD process.

An essential element to understanding the process is therefore the development and validation of molecular tools to monitor the community structure at these various process stages. Molecular indicators of ATAD diversity can be achieved by the coupling of PCR amplification of taxonomic markers with DGGE to determine sequence dissimilarities [42] and applying it to ATAD at various stages of the process [20]. There are a variety of tools available to compare DGGE profiles [43], which allow the recovery of several quantitative and qualitative measures of biodiversity indices. These include the Shannon diversity index that equates DGGE band intensity with species abundance [30], species richness based on number of DGGE bands and hence identifies the number of unique biomarkers within the ATAD niche at a particular time in the process. Several candidate genes can be utilized in such amplifications including the 16S rDNA gene [44], 23S rDNA [45], or the *rpoB* gene [46]. As these are universally present, they allow development and utilization of universal primers for amplification. However, these are not without their limitations as length and sequence, sequence polymorphism, specificity of primers and mismatches, co-migration of fragments together with extracted inhibitors from target extraction already discussed may all effect recovery of diversity indices [47]. Size limitations of DGGE amplicons of 100–500 bp may in some cases limit probe design and subsequent phylogenetic analysis which may be an issue where the copy state of 16S rDNA, the most commonly used biomarker in some species is high. Thus, optimization of PCR-DGGE protocols is paramount for any new niche and particularly for thermophilic niches such as ATAD.

Analysis of ATAD microorganisms at various stages of the ATAD process revealed that bacteria appeared to be the driving force in ATAD systems treating mixed domestic sludge (Table 2). Unusually, Archaea were not detected at the thermophilic stages nor were Eukarya or Fungi. The alkaline conditions and elevated temperatures, which are not conducive for Eukarya and Fungi may be a key issue in inability to detect the latter two domains. PCR-DGGE profiling using V3-V5 and V6-V8 of the 16S rDNA gene and the *rpoB* gene have been utilized to determine diversity within the system. Results indicated that the patterns recovered were dynamic, primarily as a function of the temperature shifts during processing. Many bands present at the mesophilic stages were absent at the thermophilic stages, which was indicative of adaptation, succession, and replacement as new adapted species dominate as a result of increased temperature (up to 70 °C) and pH (up to pH 8.4) [14]. Indeed, some new bands observed at the elevated temperatures were shown to migrate further down the DGGE gel reflective of sequences possessing a higher GC content. The PCR-DGGE patterns observed are overall reflective of new species emerging, potentially thermophiles with tolerance to the altered pH, but also the retention of some species from the mesophilic phase that may be reflective of a thermoduric population being retained [14]. Moving window analysis was applied to the DGGE profiles determined from DNA extracted at various stages of the ATAD process from inlet to thermophilic stage. Here, differences between profiles were plotted as a function of process stage. A shift in bacterial population richness was observed, with a decline in richness being observed at the thermophilic stage [14]. This has been interpreted as being reflective of fewer organisms within the bacterial community being able to adapt to the increased temperature and pH conditions with a unique adapted population appearing to emerge at this later stage in ATAD. Similar results have been reported for long-term studies of ATAD treating mixed domestic and meat waste where variation in PCR-DGGE profiles varied also as a function of time within a one-year sampling period [19]. It was reported that there were difficulties in excising DGGE fragments from DGGE gels; however, they utilized V3 region amplification of the PCR clone library and isolated a number of representative bands that were sequenced [19]. Sequence analysis revealed strains related to *Gemmatimonadetes, Thermus, Thermaerobacter, Nitrospira, Chloroflexi*, and many bands related to uncultured microorganisms.

## 5. Phylogenetic Analysis of the ATAD Community at the Thermophilic Stage

The ATAD process is used globally and it is well recognized that the microbial community present contributes to the degradation of total solids in ATAD sludge, to sludge ecology and to heat production [5,48]. There have been relatively few studies on the phylogeny of organisms present in ATAD reactors at the thermophilic stage. Such molecular studies have been restricted to ATAD systems treating swine waste [21] and pharmaceutical wastes [17] and interestingly some anaerobes, mainly Clostridia were identified in addition to aerobes. In terms of ATAD, treating domestic waste materials the first report focused on a waste mixture from a combined domestic activated sludge mixed with meat processing waste [19]. Here, the V3 region of bacterial and archaeal 16S rDNA was amplified from extracted DNA. No archael species were detected as confirmed later [14].

**Figure 1:**
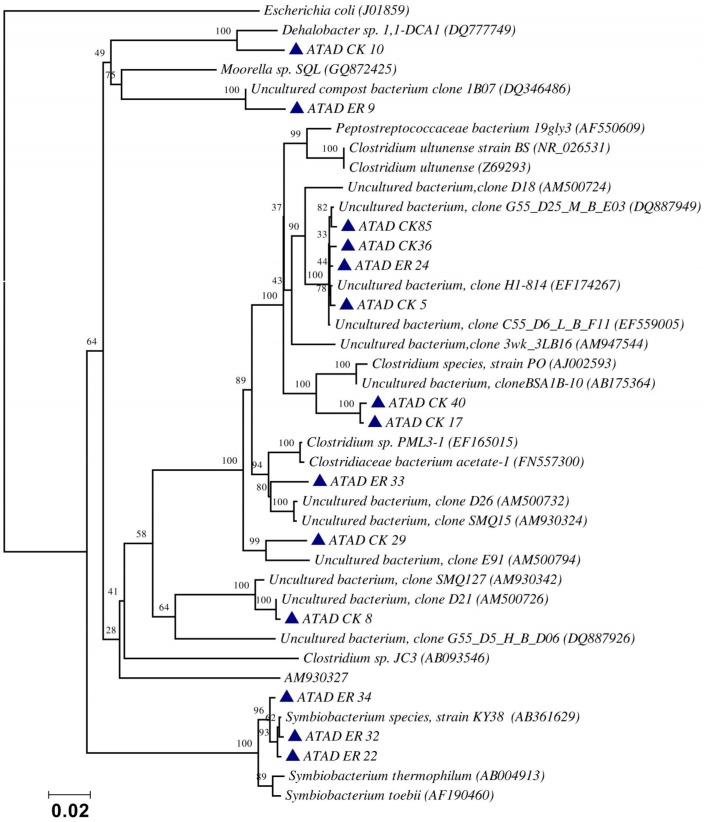
Evolutionary relationship of the ATAD clones and their closest neighbors shown as a phylogenetic tree among the Firmicutes, class Clostridia and relatives of the domain Bacteria derived by neighbor-joining analysis of 16S rDNA sequences. The bootstrap consensus tree inferred from 1000 replicates is taken to represent the evolutionary history of the taxa analyzed [51]. The percentage of replicate trees in which the associated taxa clustered together in the bootstrap test (1000 replicates) are shown above the branches. The evolutionary distances were computed using the maximum composite likelihood method and are in the units of the number of base substitutions per site. There were a total of 1238 positions in the final dataset. Phylogenetic analyses were conducted in MEGA 4.0 [52]. Reproduced with permission from Water Research, Elsevier.

**Figure 2:**
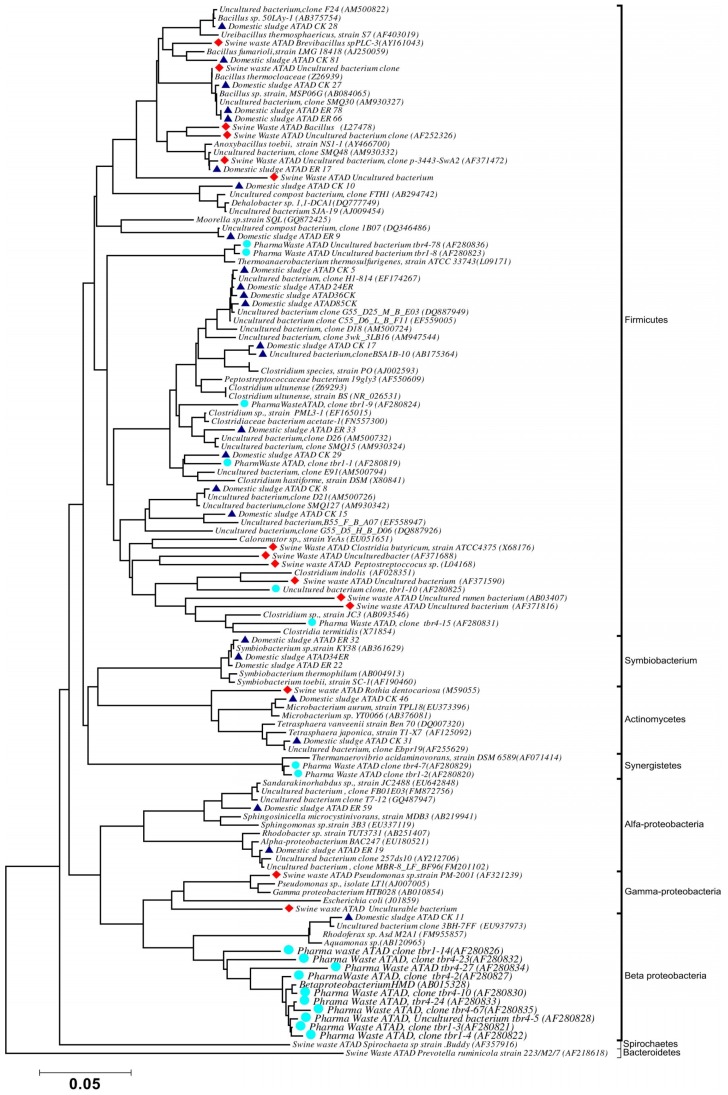
Phylogenetic positioning and evolutionary relationship of 16S rDNA clones and isolated strains obtained from ATAD reactors and their nearest neighbors Accession numbers of the sequences retrieved from GenBank are given together with their names. ATAD isolates from domestic waste [2] are illustrated with closed triangle (blue), isolates and clones from ATAD treated swine manure [21] (closed rectangular orange), isolates and clones form ATAD treated pharmaceutical waste (closed circle aquamarine) [17]. Bar scale represents 0.05 nucleotide substitutions per position. The evolutionary relationship was inferred using the neighbor-joining method, with evolutionary distances computed using the maximum composite likelihood method [53] and are in the units of number of base substitutions per site. Phylogenetic analyses were conducted in MEGA 4.0 [52]. Reproduced with permission from Water Research, Elsevier.

Using ATAD optimized extraction and purification strategies for domestic waste sludge [20,38], a comprehensive phylogenetic study was carried out on an ATAD treating mixed domestic waste sludge [2]. To limit PCR bias, amplification was carried our using universal primers 27F and 1492R to amplify almost the complete 16S rDNA gene using hot start, low cycle number with five replicas and utilizing touchdown PCR [49]. These were purified and used to generate a comprehensive clone library in the pGEM-T Easy vector (Promega, Southampton, UK), Clones demonstrating unique patterns via Amplified rDNA Restriction Analysis (ARDRA) were chosen, examined by DGGE and those deemed unique were reamplified using V3-V5 region primers. These unique sequences representing optimal ATAD diversity were sequenced and utilized for bioinformatics analysis [2]. All rDNA sequences obtained were deposited in the GenBank database under accession numbers GU376459-GU376467, GU348989, GU325824-GU325838, GU320653-GU320666, and GU437223-GU437232. Some 400 clones were examined with some 27 operational taxonomic units (OTUs) identified by ARDRA analysis. Fifty-four near full-length 16S rDNA amplicons, two each for each of the 27 OTUs were utilized. Most of the resulting clones demonstrated >92% sequence identity to deposited sequences in the Ribosomal Database. Representatives of High GC Actinobacteria, low GC Gram-positive Firmicutes, as well as α and β- Proteobacteria were observed. 22 of the phylotypes detected grouped within the Firmicutes with some fifteen of the Phylotypes representing *Clostridia* species indicating that although termed ‘aerobic’ significant anaerobic metabolism may be a feature of ATAD, perhaps due to poor mixing, poor oxygen solubility at elevated temperatures and the rate of oxygen utilization at elevated temperatures (Figure 1). Many clones demonstrated similarity to compost related uncultured organisms, which had previously been reported [17,19] from other ATAD studies. The predominance of the Firmicutes was identified in all studies and is also a feature of later phylogenetic studies [3]. Similarity to known organisms was demonstrated [14] with *Bacillus thermocloaceae* 98% (clone ER66), Anoxybacillus 99% (clone ER17), to Ureibacillus 99% (clone F27) and the alkaline tolerant *Bacillus infernus* 99% (clone CK81). ATAD Clones ER33, CK36, CK85, CK40, CK17, CK3, CK34, CK29, and CK8 showed between 96–99% similarity to cultured and uncultured Clostridia species. Clone ER22 and ER34 showed similarity to Symbiobacterium, clone ER9 to Moorella, while clone CK10 showed similarity to a *Dehalobacter* species. Clone ER19 showed 97% similarity to *Rhodobacter*, while clone ER58 showed similarity to *Sphingomonadaceae* both belonging to the α-Proteobacteriae. Clone CK11 was related to *Aquamonas* belonging to the β-Proteobacteria. Two phylotypes, CK46 and CK31, showed similarity to Actinobacteria (*Microbacterium* and *Tetrashpaera* respectively). No clones related to *Thermus* were recovered in this study as reported elsewhere [19,50] but this may reflect differences in the waste as a substrate source, the reactor design, or the operating parameters (Figure 2).

Using a Japanese ATAD as a model, Tashiro et al. [3] carried out principal-coordinate analysis of 16S rDNA amplicons from some 454,122 sequencing reads from 20 different sampling regimes during the ATAD process. They reported a predominance of Bacteroides and Firmicutes with Proteobacteria showing a dramatic increase as the process initiated. As the process increased in temperature Bacteroides numbers increased while Proteobacteria decreased. As the temperature increased towards the thermophilic stage Acinobacteria increased and the Firmicutes became the dominant phylum [3]. This unique limited species similarity was also illustrated in an examination of an ATAD generating liquid fertilizer in Japan where during the thermophilic phase a stable bacterial community was observed with major OTUs showing limited similarities to *Heliorestis baculata*, *Caldicellulosiruptor bescii*, and *Ornatilinea apprima* [23]. As with previous studies, the assignment to nearest genera showed only 84% similarity indicative that many of the bacterial species have so far not been isolated or identified. This picture is similar to previous studies [2,12,17,19,50] where OTU assignment to closest relative ranged from between 80–95% for many of the 16S rDNA species detected. This is suggestive that there are many new species to be yet identified and cultured, particularly at the thermophilic stages. Because many of these species may be thermophilic or thermoduric, live at high population densities and in at alkaline pH there are biotechnological opportunities to discover potential antibiotics (which may control access to substrate at dense populations) and thermostable enzymes with unique specificities. These may be able to utilize more recalcitrant substrates present during the late thermophilic stages with potentially a wider spectra of pH tolerances.

Although it may be impossible to remove all bias, strategies to optimize molecular approaches for determining ATAD community structure at elevated temperatures have aided in recovering this diversity [2,3,17,19,21]. The data suggests a restricted phyla distribution but a richness in members of the Firmicutes, suggesting an important role for them in ATAD digestion. However data suggests that ATAD community structure may reflect unique players depending on the chemical composition of the waste being treated and the process operating conditions with variation observed depending on the waste whether swine waste [21], pharmaceutical waste [17], meat waste [19], or mixed domestic waste [2]. While no Bacillaceae were detected in pharmaceutical ATAD waste, these were found to be particularly rich in all other studies reported. The presence of anaerobes also appears to be important with anaerobes related to *Clostridia* being detected in all published studies. However, their detection does not necessarily mean that they are active metabolically, a question that needs to be addressed in future studies.

## 6. Elimination of Pathogens

One of the key issues with the ATAD process is the provision of a stabilized sludge free from pathogens. Many studies have revealed that the combination of process temperature achieved, >60 °C and up to 75 °C on occasion, combined to holding time >254 h produces a pathogen free sludge. The absence of pathogens has been determined by both culture and culture free molecular techniques [3,9,54]. Although thermal inactivation undoubtedly plays a role, it has also been reported that cell-lysis activities towards gram-negatives—but not gram positives—may play a key role also in activating many pathogens [23].

An energy model for an ATAD treating domestic sludge has been developed and validated [55] with the principle heat source being microbial oxidation. The estimation was 1.190 MJ hr^−1^t^−2^ with the heating potential being estimated based on chemical oxygen demand (COD) at 12.1 kJ g^−1^. Although heat loss from poor reactor design and evaporation plays a role-improved design could maximize operation and potentially lead to high levels of heat recovery and improved pasteurization.

## 7. Metabolic Drivers of the Autothermal Niche

A key question for the ATAD reaction is what is driving the metabolic activity, particularly at the autothermal thermophilic stage. Microscopic analysis of the sludge [33] has revealed that a dynamic sludge morphology occurs. At the early feed stages of the process, large amounts of fiber are observed as might be expected following primary and secondary treatments of domestic waste. As the auto heating stage began during the mesophilic stage, this fibrous material remained largely intact and only showed reduction as the temperatures approached 59 °C during this phase the fibrous material underwent a major morphological change in terms of decreased fragment length and solubility [33]. In addition, the fibrous material became bulky, swollen, agglomerating, and eroding—indicative of it undergoing degradation. This has been suggested as a major driver of the thermophilic stage when the more readily utilizable substrates have been exhausted. The sludge material was also fractionated to examine the biopolymer content as a function of ATAD stage and optical sectioning, multiple staining, and laser scanning confocal microscopy (LSCM) used to examine the special distribution of the various types of biopolymer present [33]. During the thermophilic stages, the bulk water associated with the samples contained emulsified lipid droplets. Large amounts of thermostable esterase activity was also detected at this stage, suggesting these droplets may serve as a source of nutrients also [33] with many esterases being shown to be thermostable and function at elevated pH [56]. LSCM also revealed the presence of large quantities of bacteria associated with ATAD particles at the thermophilic stage. The interface between the particles and the bulk water was high also in bacterial numbers. Many of the particles contained a labyrinth of channels with β-carbohydrate and protein shells forming a hydrophobic coat. This appeared to provide a surface for the formation of microbial aggregates similar to biofilm at these particle surfaces [33]. 4′6-diamidino-2-phenylindole (DAPI) was used to enumerate bacterial counts [57] in the ATAD sludge, using random fields and duplicates at various stages under UV via epifluorescence microscopy [33]. At inlet, bacterial counts as enumerated by DAPI staining were 7.3 ± 0.4 × 10^8^, this rose sharply to 9.2 ± 0.1 × 10^13^ upon aeration and temperature increase in the mesophilic reactor. This number dropped to 6.2 ± 0.2 × 10^10^ as the reactor transitioned to the thermophilic stage but rose again following some 20 h to 5.2 ± 0.12 × 10^12^ at the late thermophilic stage when the reactor was 65 °C. These observations indicate that a thermophilic population is actively metabolizing the sludge at the thermophilic stage and although the species richness is somewhat limited, the numbers of active metabolizers is high [33].

Given that the organic fraction of ATAD sludge is the driving force for metabolically active heat-producing microorganisms, the nature of this material and the enzymatic capabilities of the microbial population are key to the success of the process. Thus, monitoring the organic matter of the sludge as the process proceeds is a key component in attempting to understand the drivers of the autothermal stage. Numerous studies have reported the initial production and utilization of volatile fatty acids during the ATAD process but these are usually substrates during the initial phases [3]. Organisms such as *Acinetobacter* spp are known to be active consumers of volatile fatty acids and have been reported in the initial stages of ATAD digestion [3]. The pattern of chemical changes and biodegradation occurring in the macromolecular fraction during ATAD digestion of domestic waste has also been examined by solid-state ^13^C NMR CP-MAS [58]. This technique is sensitive [59,60], non-destructive, independent of sample solubility and can be performed at various temperatures so is ideal to monitor chemical changes in ATAD sludge. Several observations were made. There was a reduction in total solids from 6.3% to 4.2% on average during the process, and a reduction in volatile solids of 39.2%. Feed color was also noted to change from grey to a brown color presumably because of Maillard condensation [61] of amino groups with carbohydrates. During the self-heating stage an increase in the O-alkyl fraction +16.2%) was observed with a decline in the alkyl region (−14.2%) with higher degradation rates occurring at the thermophilic stage, no difference in the carboxy-C domain was observable. The O-alkyl increase has been attributed to accumulation of soluble products of cellulose rich material, which may lose its crystallinity (an observation supported by staining and microscopic analysis Piterina et al. [33] due to release of ammonia from proteinaceous material and the consequent increase in pH to >pH 8. The initial ATAD pH at the mesophilic stage is below 7.0 (around 6.6). As deamination begins the release of ammonia from proteinaceous material begins a pH rise. As there is little buffering the pH rises to >pH 8 during the thermophilic stage [7,8,9]. Cellulolytic *Bacillus* and *Clostridia* species observed in the phylogenetic analysis at elevated temperatures may thus be responsible for this and may contribute to the O-alkyl fractions due to their cell wall components and the increase in microbial numbers [2,56]. Protein and lipids decline during the overall process due to proteolytic and esterase activities detected at elevated temperatures. A low intensity aromatic region corresponding to lignins, phenols, amine, and aromatic ethers was detected in the ATAD samples and showed a decrease (11–7.8%) indicating some contribution of these moieties to the over autothermal process. However, in many cases although spectral differences can be observed large increase in biomass detected may also be a contributing factor to the spectral changes observed by NMR.

The low index of hydrophobicity and low ratio of aliphatic compounds to carbonaceous compound detected in the 13C-NMR analysis in ATAD sludge may be important in predicting floc dissociation and may in fact be a reason why ATAD sludge has extremely poor settleability [62,63]. This poor settleability has limited somewhat the use of ATAD biosolids as with only 4.5% TS in the final product the lack of settleability means that more than 95% of the product is water, which is an issue with transport costs of the treated sludge. The pH rise observed during the ATAD process has been attributed to the release of ammonia from proteinaceous degradation during the process. In several ATAD systems, this ammonia is released and subsequently recovered [7]. No denitrifying species have been reported in ATAD systems and ammonia nitrogen levels have been reported to be high at all stages of the process [3]. This high ammonia nitrogen level suggests that stabilized ATAD sludge will have excellent liquid fertilizer potential.

## 8. Conclusions

ATAD is a tertiary treatment process to stabilize waste sludge from a variety of sources. The process carried out in jacketed reactors with aeration results in a unique thermophilic microbial community capable of generating heat, reducing the total solids of the sludge and producing Class A Biosolids with some fertilizer potential. Tools for molecular analysis of the microbial community have demonstrated it to be dynamic and responsive to the changes in temperature and pH with the community adapting to the ATAD niche. The nature of the biodegradation processes may mean that the unique thermophilic population possesses a unique arsenal of enzymes and biodegradative capabilities, which have yet not been exploited.

One of the limitations of widespread use of ATAD is the energy cost of aeration and mixing [3,8]. However, this cost could be reduced significantly by utilizing heat recovery processes [64] from the heated ATAD sludge and by decreasing the treatment time, perhaps by using the heat generated to decrease the autothermal phase.

## Figures and Tables

**Table 1:** Types of waste materials treated by ATAD processes (data adapted from [5]).

Waste Type	Reference
Livestock manure	[11]
Piggery waste	[12]
Poultry litter	[13]
Domestic municipal waste	[14]
Slaughter house wastes	[15]
Brewery waste	[16]
Pharmaceutical wastes	[17]
Paper waste	[18]
Food waste	[4]
Fermentation waste	[5]

**Table 2:** Determination of species diversity associated with ATAD sludge at various stages of the ATAD process from sludge inlet of new sludge material through reactor 1 operating at 40 °C, through reactor 2 operating at 70 °C to treated sludge storage.

DNA Extraction	Eukarya	Archaea	Bacteria	Fungi
Sludge inlet	+	+	+	+
Reactor 1 40 °C	−	−	+	+
Reactor 2 70 °C	−	−	+	−
Sludge storage day 1	−	−	+	−
